# Synthesis and Evaluation of 3D Nitrogen Doped Reduced Graphene Oxide (3D N@rGO) Macrostructure for Boosted Solar Driven Interfacial Desalination of Saline Water

**DOI:** 10.1002/gch2.202400080

**Published:** 2025-02-25

**Authors:** Fisseha A Bezza, Samuel A. Iwarere, Shepherd M. Tichapondwa, Hendrik G. Brink, Michael O. Daramola, Evans MN Chirwa

**Affiliations:** ^1^ Sustainable Energy and Environment Research Group Department of Chemical Engineering University of Pretoria Pretoria 0002 South Africa

**Keywords:** interfacial desalination, nitrogen‐doped, photothermal conversion efficiency, photothermal material, reduced graphene oxide

## Abstract

Recently, there has been a growing interest in solar‐driven interfacial desalination technology, which focuses on the localization of heat to the air‐water interface. In this study, 3D nitrogen‐doped reduced graphene oxide (3D N@rGO) photothermal material is synthesized with a facile one‐step hydrothermal process. The material exhibited richer porosity, high hydrophilicity for efficient water channeling, and all‐directional solar absorption potential. The 3D N@rGO solar absorber attained up to ≈55 °C surface temperature rise and showed ≈134% photothermal conversion efficiency with 1.94 kg m^−2^ h^−1^ net freshwater generation rate under 1 sun solar illumination, owing to efficient latent heat recycle. On a high salinity desalination study performed using 10 and 20 wt.% salinity levels, the photothermal material showed 1.66 and 1.31 kg m^−2^ h^−1^ evaporation rates respectively. It sustained stable long‐term desalination performance without visible salt accumulation on the surface up to a salinity level of 10 wt.%. In a three‐day outdoor test carried out utilizing simulated seawater with a 3.5 wt.% NaCl solution, the 3D evaporator demonstrated an average freshwater production rate of 2.61 kg m^−2^ h^−1^, during the test the solar power density reached up to 1.1 kW m^−2^. The 3D solar absorber exhibited a promising potential for large‐scale seawater desalination in water‐scarce regions worldwide.

## Graphical abstract

In this study, a novel 3D N@rGO solar absorber is fabricated, exhibiting broadband solar absorption across the spectrum and superior photothermal conversion efficiency. Under a solar‐driven interfacial desalination setup with an efficient heat management system, the 3D solar absorber demonstrated outstanding long‐term and stable desalination performance of high‐salinity water under both simulated and real‐world conditions, indicating its potential for scaling up.

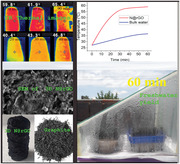

## 1 Introduction

Freshwater shortage is one of the most critical challenges of the modern world today. While nearly three‐fourths of the earth's surface is covered with water, only 3% is freshwater from groundwater aquifers, rivers, and lakes, the remaining ≈97% exists as saline water in oceans and seas making it undrinkable as well as unusable for the majority of domestic and commercial uses.^[^ ^1^ ^]^ In recent years, desalination technologies have garnered significant attention as a solution to the escalating freshwater crisis, enabling the extraction of fresh water from virtually unlimited saline sources such as seas, oceans, and brackish groundwater.^[^ ^2^ ^]^ However, conventional desalination methods, including membrane‐based techniques like reverse osmosis (RO) and electrodialysis (ED), as well as thermal‐based processes such as multi‐stage flash (MSF) and multi‐effect distillation (MED), are known for their high energy demands and substantial installation and operational costs.^[^ ^3^ ^]^

A promising alternative that has recently emerged is solar‐driven interfacial desalination. This approach concentrates heat at the water‐air interface, reducing heat loss to the bulk non‐evaporative liquid and achieving high photothermal conversion efficiency.^[^ ^4^ ^]^ Unlike traditional solar desalination methods, which require significant energy input, this innovative technique enables nanostructured evaporators to enhance photothermal conversion efficiency. By positioning a porous solar absorber on the water's surface, interfacial solar‐to‐heat conversion is achieved without the need for complex pressure controls or costly infrastructure. The solar energy is focused on the absorber's surface, creating high temperatures that facilitate efficient heat transfer to the surrounding liquid. This results in rapid heating of the water, triggering phase change and vapor generation.

What sets solar‐driven interfacial desalination apart is the strategic placement of the solar absorber at the interface between the saline water and the air, which minimizes heat loss to the bulk liquid while providing an extensive surface area for effective vapor release.^[^ ^5^ ^]^ To ensure optimal performance, an interfacial desalination system must exhibit superior thermal management, effective heat localization with low thermal conductivity, efficient water transfer to the evaporative surface via capillary forces, excellent solar‐thermal conversion efficiency, high resistance to salt accumulation, long‐term operational stability, and scalability for production.^[^ ^3^, ^5^ ^]^ The performance of this technology primarily hinges on the solar absorber's ability to absorb light and convert it into heat efficiently. A key characteristic of high‐performing photothermal materials is their capacity for broadband light absorption across the solar spectrum, spanning wavelengths from 200 to 2500 nm at sea level.^[^ ^6^ ^]^

Graphene, a single layer of carbon atoms arranged in a 2D honeycomb lattice, has attracted immense interest in both basic and applied research due to its extraordinary physical and chemical properties. These include a high theoretical surface area of ≈2630 m^2^ g⁻¹, exceptional chemical stability, superior electrical conductivity (106 S cm⁻¹, exceeding that of silver, the most conductive material at room temperature), a unique graphitic basal plane structure, and its ease of functionalization and scalable production. These exceptional attributes make graphene an ideal candidate for designing solar absorbers.^[^ ^7^ ^]^ Additionally, graphene is a semi‐metal with zero bandgaps, the absence of a bandgap enables it to absorb electromagnetic radiation over a very wide bandwidth.^[^ ^8^ ^]^ However, pristine 2D graphene nanosheets are incapable of forming stable homogenous dispersions as a result of their hydrophobicity and suffer from aggregation or severe face‐to‐face restacking of graphene sheets in solution due to van der Waals interactions, making their distinctive intrinsic properties less effective, leading to less efficiency than the theoretical values.^[^ ^9^ ^]^ A highly effective method for addressing this issue involves the reconstruction of 2D layers into meticulously arranged and interconnected 3D structures. This approach not only preserves the remarkable characteristics of 2D graphene materials but also allows for their convenient utilization without the concern of restacking.^[^ ^7^, ^10^ ^]^

The formation of interconnected and porous 3D reduced graphene‐based structures from the 2D graphene sheets confers adequate hydrophilicity to the 3D interconnected structure for efficient interfacial water desalination purposes while retaining the exceptional properties of the 2D graphene.^[^ ^10^ ^]^ Different methods have been employed to produce porous 3D frameworks using graphene oxide (GO), including hydrothermal reduction, chemical reduction, laser casting, and 3D printing.^[^ ^7^ ^]^ The conversion of graphite to GO followed by its  tailored reduction plays a crucial role in producing a porous 3D reduced graphene oxide (3D rGO) material with a well‐controlled structure and properties.^[^ ^7^ ^]^ The interconnected 3D rGO network structures are of great scientific interest due to their retention of graphene's exceptional physical characteristics, such as high electrical and thermal conductivity, as well as remarkable mechanical and chemical stability. Additionally, the 3D rGO provides a hydrophilic surface for easy capillary force driven transport of water, ensuring adequate  permeation of the evaporator's surface on account of its rich  oxygen containing functional groups that enhance hydrophilicity and dispersibility.^[^ ^9^ ^]^

Furthermore, the introduction of heteroatoms like boron (B), nitrogen (N), phosphorus (P), sulfur (S), bromine (Br), and iodine (I) via chemical doping is a fundamental technique for tailoring the electronic properties and chemical reactivity of graphene. This is due to the significant differences in size and electronegativity between heteroatoms and carbon atoms. The incorporation of heteroatoms results in the creation of novel functionalities for graphene, broadening its potential applications.^[^ ^11^ ^]^ Extensive research has been conducted to produce diverse heteroatom‐doped graphene materials and graphene‐based nanocomposites to fully harness their outstanding characteristics in various fields, including solar energy conversion and energy storage systems.^[^ ^12^ ^]^ Particularly, nitrogen doping plays a critical role in adjusting the electronic and chemical properties of carbon materials, owing to its comparable atomic size and ability to form strong bonds with carbon atoms through its five valence electrons.^[^ ^11^, ^13^ ^]^ The introduction of dopants can impart new functionalities to the 3D rGO materials and render them more capable of efficient photothermal conversion and steam generation.^[^ ^14^ ^]^ In this work, 3D porous nitrogen‐doped reduced graphene oxide was synthesized, and its photothermal conversion efficiency and saline water desalination potential were investigated.

## 2 Experimental Section

### 2.1 Preparation of 3D N@rGO

#### 2.1.1 Synthesis of GO

GO was synthesized following Tour's method as described by Habte and Ayele.^[^ ^15^ ^]^ Briefly, a solution was prepared by mixing 360 mL of concentrated sulfuric acid (H₂SO₄) and 40 mL of concentrated ortho‐phosphoric acid (H₃PO₄) in a 9:1 volumetric ratio. This solution was then added to a mixture of 3.0 g of graphite powder and 18 g of potassium permanganate (KMnO₄), causing the solution temperature to rise to 40–45 °C. The mixture was heated to 50 °C in a temperature‐controlled water bath and stirred continuously for 24 h, during which it gradually developed a pasty, muddy consistency. After 24 h, 400 mL of ice water was added to the mixture, followed by 30 mL of 30 wt.% hydrogen peroxide (H₂O₂) to reduce manganese ions (MnO₄⁻) into soluble manganese sulfate and manganese oxide (MnO₂), effectively halting the reaction. The addition of H₂O₂ caused bubbling and a bright yellow coloration, signifying substantial oxidation. The GO was then collected by centrifugation (10 min at 8000 rpm) and thoroughly washed with 200 mL of 30% hydrochloric acid (HCl) to remove any residual sulfate ions. The resulting GO was subsequently used for the synthesis of 3D N@rGO. 

#### 2.1.2 Synthesis of 3D N@rGO 

3D N@rGO was synthesized using a hydrothermal self‐assembly technique. Briefly, the GO was dispersed in deionized water via ultrasonication, producing a 100 mL GO dispersion with a concentration of 45 mg mL^−1^. Subsequently, 5 g of ethylenediaminetetraacetic acid (EDTA) was added to the dispersion to provide a nitrogen source, and the mixture was further ultrasonicated for 30 min to achieve a homogeneous dispersion. EDTA, a highly basic amine with a nitrogen content of 46.6 wt.%, served as the nitrogen precursor for doping graphene oxide. The homogeneous dispersion solution was then transferred to a 300 mL Teflon‐lined stainless‐steel autoclave, where hydrothermal reduction was carried out at 180 °C for 18 h. Following this, the self‐assembled 3D N@rGO structure was carefully taken out from the autoclave and subsequently frozen at −80 °C. Freeze drying was then performed for 60 h to obtain porous and structurally robust 3D N@rGO.^[^ ^16^ ^]^

### 2.2 Characterization

High‐field emission scanning electron microscopy (FESEM) coupled with energy dispersive X‐ray (EDX) analysis was employed to investigate the morphology and elemental composition of the synthesized samples. These analyses were performed using FESEM and energy‐dispersive X‐ray spectroscopy (EDS, JEOL‐7800F, JEOL). The broadband diffused solar absorption capabilities of the GO and rGO samples were evaluated over a wide wavelength range (300–2500 nm) and at various angles using a UV–vis–NIR spectrophotometer (UV3600, Shimadzu Scientific Instruments).

X‐ray diffraction (XRD) analysis was conducted to assess the crystallinity and phase purity of the samples. The measurements utilized Cu‐Kα radiation (λ = 1.540 Å) over a 2θ range of 5°–90°, with a step size of 0.05° s^−1^. Fourier Transform Infrared (FTIR) spectroscopy was employed to identify functional groups within the samples. The FTIR spectra were recorded under ambient conditions using a Perkin‐Elmer FTIR spectrometer (Model Spectrum 100 Series, USA) in the wavenumber range of 4000–400 cm⁻¹, with a resolution of 4 cm⁻¹.

X‐ray photoelectron spectroscopy (XPS) was performed using a Thermo ESCALAB 250Xi spectrometer, equipped with a monochromatic Al Kα source (1486.7 eV) operating at 300 W. The X‐ray spot size was set to 900 µm, and the chamber pressure was maintained at <10⁻⁸ mBar. Survey spectra were acquired at a pass energy of 100 eV, while high‐resolution spectra, focusing on the C 1s, O 1s, and N 1s core levels of nitrogen‐doped carbon (N─C) materials, were recorded at a pass energy of 20 eV.

### 2.3 Solar‐Driven Interfacial Evaporation Performance Test

**Figure 1:**
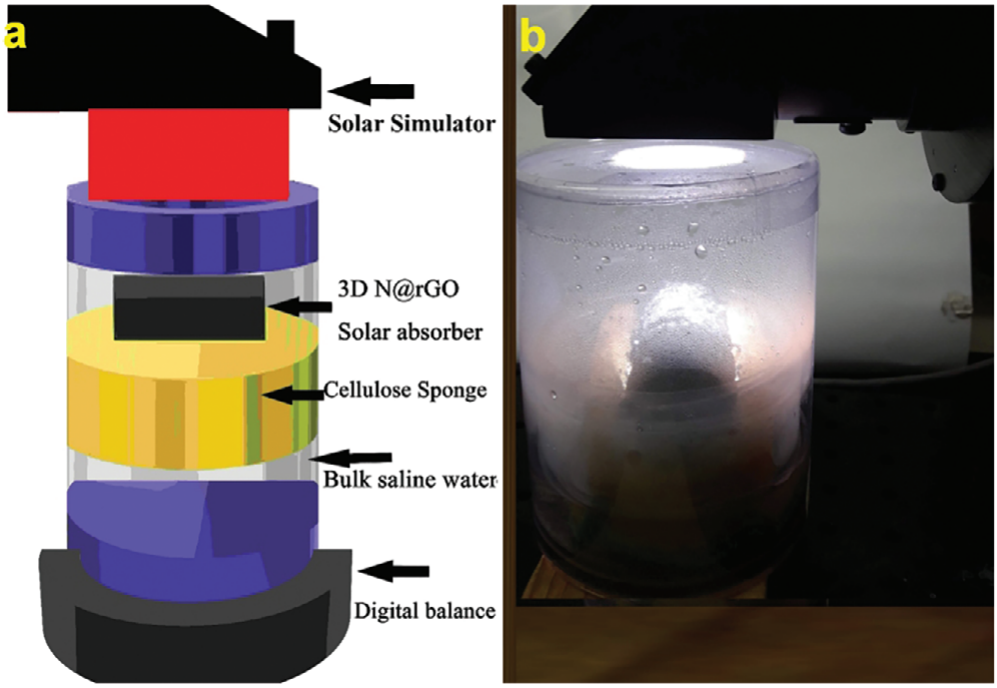
Schematic illustration of the experimental interfacial desalination setup a), coupled with the corresponding digital photograph b). The setup consists of the 3D N@rGO photothermal material placed on the cellulose sponge, which is in turn placed on top of the hypersaline water. The entire setup is enclosed within a glass enclosure, which serves two purposes: it acts as the condensation area for the vapor generated and provides insulation for the heat generated.

To investigate the solar‐driven interfacial evaporation performance of the 3D N@rGO, a setup using the 3D N@rGO as the light absorber, a beaker filled with the water supply, and a solar simulator as an energy source was constructed as displayed in **Figure** **1**. The 3D hierarchically porous evaporator was placed on a cellulose sponge. The cellulose sponge between the solar evaporator and the bulk water is used as a thermal insulation medium, and as a water transfer channel to avoid heat dissipation into the bulk water. The cellulose sponge was chosen as the substrate material, as it generally provides the best water transport/evaporation and thermal insulation owing to its low thermal conductivity and porous structures. It increases the thermal resistance between the absorber and bulk water, which increases the photothermal‐steam conversion efficiency.^[^ ^17^ ^]^ The solar‐driven interfacial desalination study was conducted at a temperature of 27.0 ± 0.5 °C. A solar simulator (Xe lamp, PL‐X300) was used as the source of solar energy in the indoor tests and an electronic balance was used to accurately measure the mass of water produced per unit effective surface area of the 3D solar absorber. The evaporation area was determined to be the top surface of the evaporator directly exposed to the irradiated light (≈7 cm × 4 cm = 28 cm^2^ = 0.0028 m^2^ when placed horizontally or ≈π × (2cm)^2^ = 0.00125664 m^2^, when placed vertically). Long‐term solar‐driven interfacial desalination performance and stability of the 3D N@rGO solar absorber were studied via exposure to simulated solar light for three cycles, each lasting 7 h. The evaporation rate was determined by observing the mass change of the reservoir under one sun illumination and in darkness.

The potential of the 3D evaporator in desalinating hypersaline water was evaluated through the utilization of saline water with increasing salinity (3.5, 10, 20 wt.%) for three cycles lasting 9 h each. Additionally, the self‐cleaning ability of the 3D evaporator was assessed by introducing 2 grams of salt onto its surface during the solar‐driven desalination process.

#### 2.3.1 Real‐World Application Potential and Long‐Term Performance Test

The real‐world application potential of the 3D N@rGO and its long‐term stability to desalinate saline water over an extended duration, as well as its salt resistance potential, were evaluated over the course of 3 days, under 8 h of irradiation each day on a rooftop. The surface temperature of the 3D N@rGO evaporator was measured periodically using a FLIR TG165 infrared camera.

## 3 Results and Discussion

### 3.1 Morphological and Optical Properties

**Figure 2:**
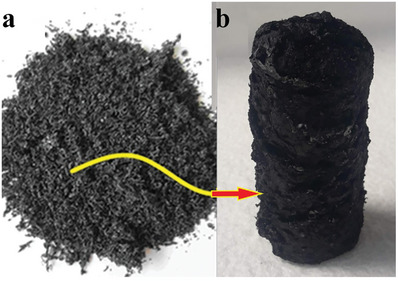
Pristine graphite sheets a); oxidized and used for the synthesis of hydrothermally treated 3D N@rGO macroporous solar absorber b).

**Figure** **2b** shows the 3D N@rGO macroporous solar absorber synthesized through hydrothermal reduction of GO and ethylenediamine (EDTA) homogeneous dispersion. Owing to the easily dispersible character of GO conferred by the oxygen functional groups like hydroxyl, epoxide, and carboxylic at the basal planes, the graphene oxide sheets interconnect through covalent bonding and form a 3D interconnected network in the hydrothermal reduction process.

**Figure 3:**
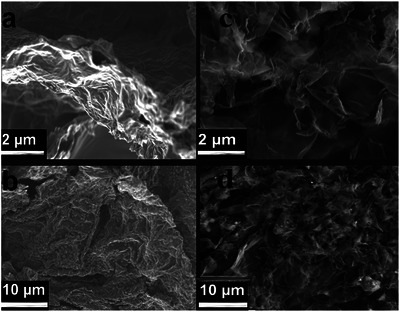
SEM micrographs of rGO a, b) and N@rGO c, d) exhibiting porous and wrinkled morphologies at different magnifications.

As illustrated in **Figure** **3**, the SEM images reveal the porous and wrinkled morphology evident in both the reduced graphene oxide (rGO), Figure 3a,b, and N@rGO, Figure 3c,d at different magnifications. While both rGO and N@rGO exhibit fluffy and highly wrinkled morphologies, indicative of their high porous structure and large surface area that favors increased water and heat absorption potential, a closer examination of their morphologies reveals distinct characteristics. In comparison to the morphology of rGO, the N@rGO consists of entangled and severely crumpled sheets closely associated with each other and forming disordered solid, multilayer structures. This observed difference may be attributed to the defects introduced on to graphene by the nitrogen atoms doping. Nitrogen doping has been reported to induce structural defects, more active sites increased electrical conductivity, ultrahigh electron mobility, and photocatalytic activity of the nanosheets.^[^ ^18^ ^]^

**Figure 4:**
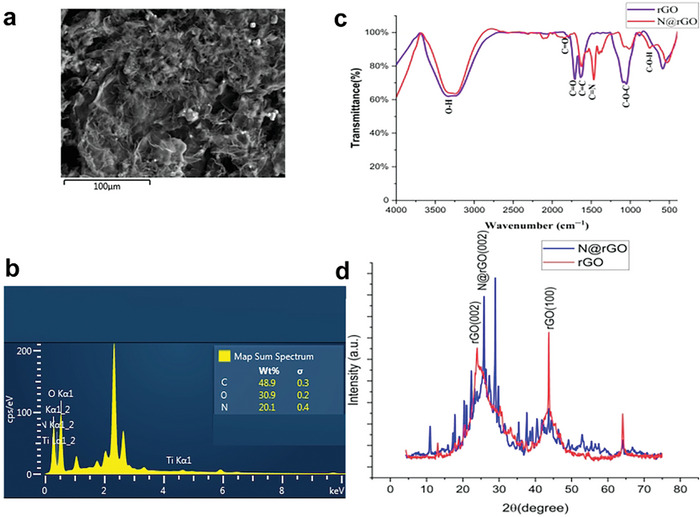
Energy‐dispersive X‐ray spectroscopy (EDX) elemental composition of the 3D N@rGO solar absorber sample a, b); FTIR spectra c) and X‐ray diffraction (XRD) pattern of rGO and N@rGO d).

The wrinkled and crumpled structure of N@rGO observed in the SEM images enhances light absorption through the multiple reflection effect and facilitates water transfer via interconnected pores. The Energy‐dispersive X‐ray spectroscopy (EDX) elemental mapping of the N@rGO sample (**Figure** **4**a,b), revealed a significant amount of nitrogen incorporated into the composite.

The FTIR peaks shown in Figure 4c display distinct bands in both rGO and N@rGO corresponding to functional groups such as epoxy (C─O) at ≈1100 cm⁻¹, carbonyl (C═O) at ≈1745 cm⁻¹, hydroxyl (─OH) ≈3350 cm⁻¹.^[^ ^19^, ^20^, ^21^ ^]^ The peak located at 1635 cm⁻¹ can be ascribed to the skeletal vibration of graphitic domains. For the N@rGO, in addition to the aforementioned peaks, the peak ≈1447 cm⁻¹ is associated with the C═N stretching (carbon═nitrogen double bond) and C─N stretching (carbon─nitrogen single bond) vibrations.^[^ ^13^, ^19^ ^]^

As shown in Figure 4d, both rGO and N@rGO display two diffraction peaks: rGO exhibits peaks at 2θ ≈23.4° and 43°, while N@rGO shows peaks at 2θ ≈25.5° and 43°. These peaks correspond to the (002) and (100) planes of the sp^2^‐hybridized graphite crystal structure.^[^ ^22^ ^]^ The respective peaks indicate the formation of graphene layers and the restoration of the sp^2^‐hybridized carbon backbone with delocalized π–π electrons after hydrothermal reduction.^[^ ^23^ ^]^ The interlayer spacing (d‐spacing) of the samples was calculated using Bragg's equation. The N@rGO sample exhibited an interlayer spacing of ≈0.348 nm, which is slightly larger than the spacing of natural graphite (0.336 nm), indicating an increase in interlayer distance for N@rGO. In contrast, rGO exhibited a prominent and broad peak at 2θ ≈23.4°, with a larger d‐spacing of ≈0.380 nm. This larger spacing is attributed to the presence of more oxygen‐containing functional groups. However, the reduction in oxygen‐containing functional groups due to nitrogen doping in N@rGO leads to a smaller interlayer spacing compared to rGO. The decreased interlayer spacing leads to more tightly packed graphene layers, improving electrical conductivity by reducing electron scattering.^[^ ^24^ ^]^

**Figure 5:**
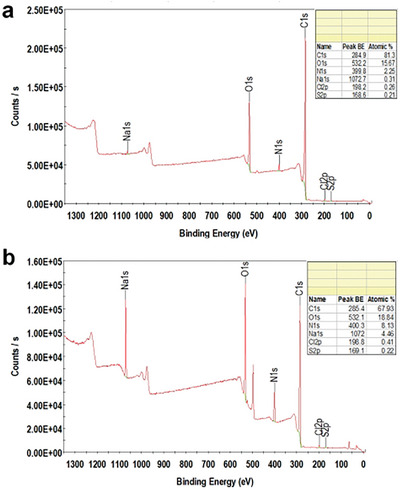
The full XPS survey scan of rGO a) and N@rGO (b), with insets highlighting the percentage composition of carbon (C), nitrogen (N), and oxygen (O) in each sample.

X‐ray photoelectron spectroscopy (XPS) was utilized to examine the nature and distribution of carbon‐based functional groups and to identify, quantify, and determine the nitrogen content within the 3D N@rGO framework. **Figure** **5**a,b present the XPS survey spectra for the undoped rGO and N‐doped rGO samples, respectively. Peaks observed at ≈285, 400, and 532 eV correspond to the C 1s (carbon‐based groups), N 1s (from doped nitrogen species), and O 1s (oxygen‐containing functional groups) present on the N@rGO and undoped rGO sheets. The undoped rGO exhibits a significantly weaker N signal, whereas the doped samples show a pronounced N peak, confirming the successful nitrogen doping of the graphene. The XPS analysis (Figure 5) revealed that EDTA treatment at 180 °C for 16 h introduced ≈8.51 at. % nitrogen into the reduced graphene oxide sheets compared to the 2.25 at.% nitrogen observed in the undoped rGO sample.

**Figure 6:**
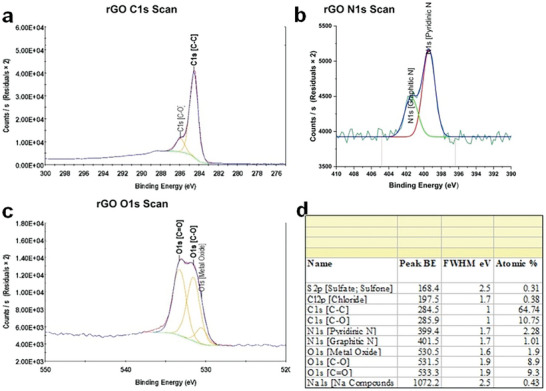
High‐resolution deconvoluted XPS spectra of a) C 1s, b) N 1s, c) O 1s, and d) their corresponding atomic percentages for rGO.

**Figure 7:**
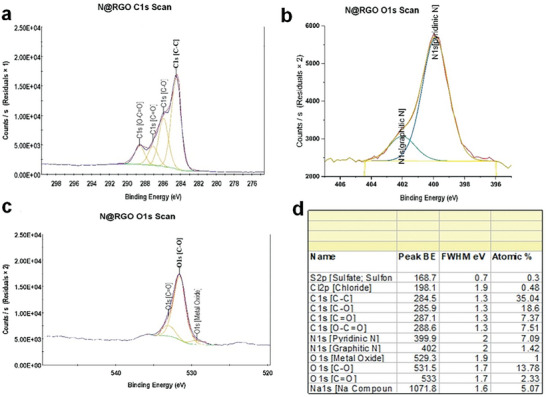
High‐resolution deconvoluted XPS spectra of a) C 1s, b) N 1s, c) O 1s, and d) their corresponding atomic percentages for N@rGO.

The N 1s spectra of rGO and N@rGO were fine‐scanned and deconvoluted to analyze the percentage and types of nitrogen species present in the samples, as illustrated in **Figures** **6**a–d and **7**a–d, respectively. When nitrogen atoms are incorporated into the graphene lattice, they typically adopt one of three bonding configurations within the carbon framework: pyridinic N, pyrrolic N, or graphitic N.^[^ ^22^, ^25^ ^]^ As illustrated in Figure 5a,b, the nitrogen peak in the XPS survey appears at ≈400 eV. Deconvolution of the N 1s peaks reveals two distinct peaks at 398.8 and 401.97 eV, corresponding to pyridinic N and graphitic N, respectively.^[^ ^26^ ^]^ A detailed deconvolution of the N 1s spectrum (Figures 6b, and 7b, with insets showing the percentage of the species) reveals significant proportions of pyridinic nitrogen (399.8 eV) accounting for 7.09 at.% and graphitic nitrogen (401.97 eV) accounting for 1.42 at.% in the N@rGO sample.

Binding energies within the range of 399.5–399.8 eV are associated with pyridinic nitrogen, where the nitrogen atom is bonded to two sp^2^‐hybridized carbon atoms in a C═N─C configuration, commonly found in aromatic rings.^[^ ^27^, ^28^, ^29^ ^]^ The pyridinic nitrogen, is indicative of the nitrogen atom's bonding to two sp^2^‐hybridized carbon atoms (C═N─C) in an aromatic ring.^[^ ^28^, ^29^ ^]^ This type of nitrogen is recognized for enhancing the electron‐rich characteristics of reduced graphene oxide (rGO), thereby improving its electronic conductivity and catalytic activity. The relatively low binding energy (BE) of pyridinic nitrogen suggests high electron density, which is facilitated by lone pairs on nitrogen, proximity to other nitrogen atoms, and resonance with the graphene π‐electron system. The peak at 401.97 eV corresponds to graphitic nitrogen, where nitrogen is incorporated into the graphene lattice, often substituting a carbon atom.^[^ ^30^ ^]^ Graphitic nitrogen further contributes to the high electron density of rGO through its interaction with the graphene π‐system.^[^ ^31^, ^32^ ^]^

The XPS survey spectra indicated carbon contents of 81 at.% and 67 at.% in rGO and N@rGO, respectively. The deconvoluted XPS spectra of the carbon component in both rGO (Figure 6a) and N@rGO (Figure 7a) display high intensities corresponding to functional groups such as carboxyl, epoxide, and hydroxyl. The sp^2^ carbon network is preserved in both rGO and N@rGO, as proven by the prominent C─C peak at 284.5 eV. Furthermore, XPS analysis reveals the presence of epoxide (285.9 eV), carbonyl (287.1 eV), and carboxyl groups in both rGO (Figure 6a,c) and N@rGO (Figure 7a,c).^[^ ^19^, ^30^, ^33^ ^]^

X‐ray photoelectron spectroscopy analysis indicated that rGO and N@rGO contained ≈15 at.% and 18 at.% oxygen, respectively. Figures 6c and 7c, along with their insets, show that the O 1s spectrum (Figure 5a,b) was separated into two main peaks at 531.5 and 533 eV, corresponding to C─O (oxidized carbon) and C═O (carbonyl or acidic functional groups), respectively.^[^ ^34^ ^]^ The XPS analysis provided essential insights into the surface composition, highlighting the substantial presence of oxygen‐containing functional groups that impact the overall performance of the solar absorber. The detailed examination identified functional groups like carboxylic, carbonyl, hydroxyl, and epoxy groups, which significantly enhance the hydrophilicity of the 3D solar absorber. This improvement in hydrophilicity facilitates efficient water transport to the evaporative surface, optimizing water flow and boosting the evaporation process.

### 3.2 Photothermal Properties of the 3D N@rGO

The optical properties of materials are directly related to the solar photothermal conversion, and the higher the light absorption, the higher the photothermal conversion capacity.^[^ ^35^ ^]^

#### 3.2.1 UV–vis–NIR Spectroscopy Analysis of N@rGO

**Figure 8:**
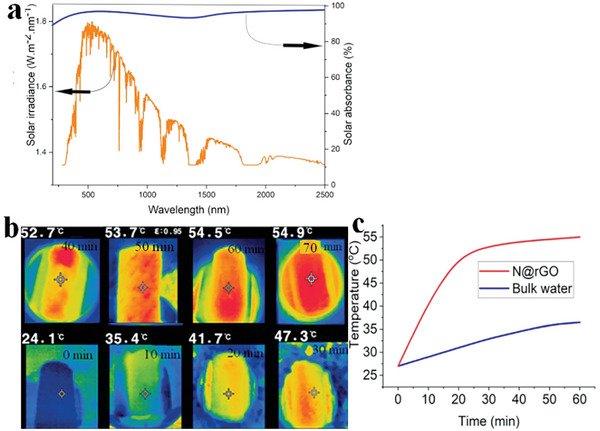
Solar absorbance of N@rGO showing broadband absorption over the broadband UV–vis–NIR region a); Infrared thermal images of the 3D N@rGO evaporator showing the temperature distribution during evaporation of 3.5 wt.% NaCl solution under one sun light irradiation monitored by an IR camera every 10 min b). Time‐dependent changes in the surface temperature of the 3D solar absorber and bulk water c).

UV–vis–NIR spectroscopy analysis of the 3D N@rGO was performed to study their photo‐absorption capacities in the range of 250–2500 nm. Efficient interfacial solar desalination mainly depends on the broadband solar absorption and Interfacial solar‐to‐heat conversion performance of solar absorbers.^[^ ^36^, ^37^, ^38^ ^]^ As presented in **Figure** **8**a. the nitrogen‐doped rGO displays outstanding solar absorption potential over the broadband UV–vis–NIR region monitored. Weighted by the AM 1.5 G solar spectrum, the broadband solar absorbance of the 3D N@rGO reached ≈97%, with minimal optical losses. This remarkable performance is attributed to the superior optical absorption properties of N@rGO, as well as the porous and rough surface structure of the material.^[^ ^39^ ^]^ The optical characteristics of the 3D rGO are improved through the incorporation of heteroatom (N‐doping), which boosts both the electrocatalytic performance and electrical conductivity of graphene. Perfect graphene with very high electrical conductivity may have a restricted number of active sites, as the electrocatalytic activity is linked to defect and edge sites.^[^ ^40^ ^]^

The doping of graphene derivatives with heteroatoms such as nitrogen (N), phosphorus (P), sulfur (S), and boron (B) effectively improves charge transfer rates. This enhancement arises from the differences in electronegativity between the heteroatoms and carbon atoms, leading to extraordinary charge polarization. This polarization, while maintaining the conjugate length of graphene, creates more chemically reactive sites.^[^ ^41^ ^]^ Nitrogen (N) doping, in particular, introduces defects and adjusts the electronic structure of graphene, enabling tailored alterations in its chemical properties. These changes make it suitable for photothermal and energy storage applications.^[^ ^42^ ^]^ Nitrogen's high electronegativity, attributed to its lone pair of unshared electrons, exerts a strong electron‐withdrawing effect when combined with carbon. This interaction enhances defects and electron flow within the graphene lattice. Nitrogen, with its five valence electrons, forms covalent bonds with rGO while retaining its lone pairs. This unique configuration improves both the electrical conductivity and chemical functionality of the material.^[^ ^43^ ^]^ N@rGO benefits from an increased electroactive surface area and enhanced conductivity due to the structural defects and electronic modifications introduced during doping, which are essential for improved electron transport and photothermal performance.^[^ ^44^, ^45^ ^]^

Nitrogen atoms donate electrons to the graphene conduction band, further enhancing electrical conductivity. These dopants create structural imperfections that result in regions of uneven electron density, thereby increasing the material's defect density, electrical conductivity, mechanical strength, and surface activity. The defects and active sites introduced by nitrogen doping expand the electrochemically active area, providing more sites for reactions. Furthermore, nitrogen atoms in N‐rGO engage in π–π stacking interactions with heteroaromatic rings, stabilizing the structure and facilitating efficient electron transport, critical for electrical conductivity and photothermal energy conversion where stability and electron transfer are paramount.^[^ ^46^, ^47^ ^]^

Additionally, the porous microstructure of N@rGO allows for quicker charge transfer and ion movement through its interconnected mesoporous and macroporous 3D networks, in contrast to rGO.^[^ ^46^, ^48^ ^]^ Nitrogen doping creates functional groups that contain both nitrogen and sulfur, which boost the material's electrochemical performance.^[^ ^49^ ^]^ The incorporation of nitrogen‐based functional groups enhances the surface wettability of rGO, improving its electrical conductivity and increasing the number of active sites.^[^ ^50^, ^51^ ^]^ Lan et al.^[^ ^50^ ^]^ demonstrated the modulation of reduced graphene oxide's surface hydrophilicity through nitrogen doping and the addition of nanoflower‐like MoS₂. This dual modification optimizes hydrophilicity, resulting in a material (MNGA) with both open and closed micrometer‐sized pores. These features allow the material to self‐float, trap air, and maximize the exposed surface area for evaporation, ensuring efficient water transport and improved photothermal performance. Nitrogen doping has also been reported to effectively modulate the hydrophilicity of graphene‐based solar absorbers, facilitating efficient water transfer to the evaporation surface.

The thermal imaging IR camera was utilized to monitor the rise in surface temperature of the 3D N@rGO during the process of interfacial evaporation, as depicted in Figure 8b. When the 3D N@rGO was subjected to 1 sun solar irradiation, the overall surface temperature of the 3D N@rGO solar absorber increased from the initial ambient temperature of 24.1 °C to a value exceeding 41.7 °C within 20 min. Subsequently, the temperature reached its maximum value of 54.5 °C and remained stable after 60 min of irradiation. Following the solar irradiation, the entire surface of the solar evaporator experienced a rapid increase in surface temperature due to its exceptional solar absorption performance. This led to the generation of a thermogradient‐driven capillary force, which facilitated the efficient flow of water from the bulk. On the other hand, as illustrated in Figure 8c, the bulk water showed a temperature increase of 9 °C from 24.1 to 36.4 °C over a time span of an hour and remained stable. In the absence of the 3D solar absorber, pure water absorbs sunlight volumetrically which increases the surface temperature only by 12.3 °C.

The high surface temperature attained in a short timespan can be attributed to the outstanding broadband solar radiation absorption potential of the 3D N@rGO evaporator, localized liquid–vapor phase change, and the porous structure of the evaporator making it suitable for efficient interfacial solar desalination application. The increase in surface temperature gives rise to a temperature gradient along the solar absorber and thus higher driving force for efficient viscous flow of water from the bulk through the hydrophilic support material.

### 3.3 Interfacial Desalination Performance of the 3D N@rGO

The 3D N@rGO exhibited broadband solar absorption and outstanding photothermal performance, superhydrophilicity, and high porous structure, which are superb properties for high photothermal conversion efficiency, adequate water supply and steam generation as well as high salt resistance potential. To evaluate the interfacial desalination performance of the 3D N@rGO, an interfacial desalination setup was prepared by placing the 3D N@rGO porous absorber on the cellulose sponge that floats on the saline water surface, under 1 sun solar illumination (1kW m^─2^), as illustrated schematically in Figure 1.

High photothermal conversion efficiency is of great importance for achieving high interfacial solar desalination performance, specifically in terms of capturing and converting as much input sunlight into heat as possible. In order to optimize the utilization of this heat for vapor generation, it is crucial to minimize heat loss through conduction, convection, and radiation. Therefore, the overall efficiency of vapor generation is determined by the combined performance of photothermal energy conversion, thermal insulation, and facilitated water transfer. The 3D N@rGO porous cylindrical evaporator, measuring 4 cm in diameter and 7 cm in length, was positioned on a cellulose sponge either horizontally or vertically. The vertical orientation was applied in the indoor solar simulated test as it yielded a comparatively higher vapor generation rate per unit area compared to the horizontal orientation, due to its smaller effective surface area. The transparent glass placed on top of the solar absorber minimized radiative heat loss and served as a condensing surface. The cellulose sponge at the bottom of the evaporator served as a thermal insulator, supporter, and water channeling material owing to its high porous structure with water channels inside.

**Figure 9:**
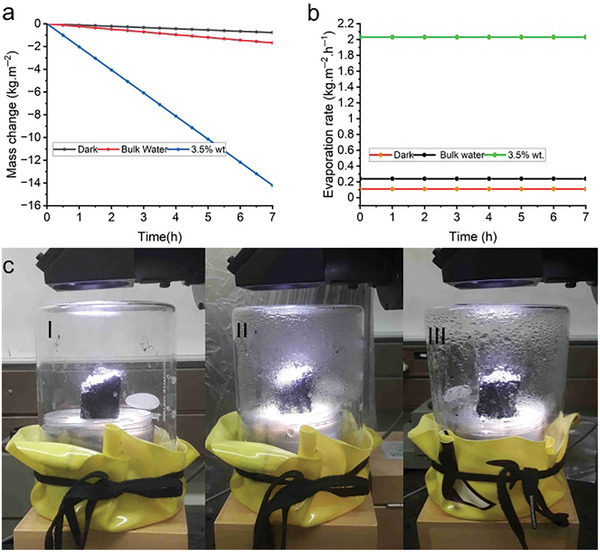
Vapor generation under 1 sun irradiation. Mass change of the simulated seawater and bulk water control under 1 sun solar irradiation as well as under dark conditions a); the corresponding evaporation rates of the saline water under 1 sun solar irradiation and dark conditions b); digital photographs of the interfacial evaporation set up c); at the beginning of the steam generation process (I), 20 min (II) and 30 mins (III) later. The setup involves the 3D N@rGO evaporator, the cellulose sponge, that supports the solar absorber and prevents the dissipation of heat into the bulk water.

The change in mass of the saline water and evaporation rate under one sun illumination and in darkness were plotted, as shown in **Figure** **9**a,b. As depicted, under 1 sun of solar irradiation, the evaporation rate increased from ≈0.24 kg m⁻^2^ h⁻¹ for bulk water, which absorbs sunlight volumetrically, to ≈2.05 kg m⁻^2^ h⁻¹ when using the 3D N@rGO photothermal material. In the absence of light, the 3D evaporator exhibited an evaporation rate of 0.11 kg m⁻^2^ h⁻¹ under similar conditions. Therefore, the net evaporation rate excluding the dark condition was ≈1.94 kg m⁻^2^ h⁻¹ under 1 sun of solar illumination. In contrast, the control experiment conducted under the same conditions but without the 3D evaporator showed a water evaporation rate of 0.24 kg m⁻^2^ h⁻¹, which is roughly one‐sixth of the amount evaporated with the 3D macrostructure. Figure 9c shows the steam generated from the surface of 3D reduced graphene oxide (3D rGO) under 1 sun solar illumination (1 kW m⁻^2^).

The efficient interfacial desalination performance of the 3D rGO can be attributed to the high hydrophilicity and water transfer potential as well as the high solar to steam conversion potential. Hydrothermal reduction of GO to rGO restores sp^2^ hybridized carbon atoms and electrical conductivity, furthermore nitrogen doping results in a higher positive charge on a carbon atom adjacent to the nitrogen atoms offering graphene a higher electrocatalytic activity and conductivity for efficient photothermal performance.^[^ ^52^ ^]^ Additionally, nitrogen doping increases the structural defects, surface area, and active cites as well as, high charge carrier potential, leading to superb photocatalytic and photothermal activity.^[^ ^53^ ^]^ In experiments conducted using undoped 3D rGO, the evaporation rate was 1.44 kg m⁻^2^ h⁻¹, which, while high, was lower than the 1.94 kg m⁻^2^ h⁻¹ achieved with nitrogen‐doped rGO in a solar‐simulated indoor test. This difference can be attributed to the enhanced properties of N@rGO, including surface defects and increased electrical conductivity induced by the electronegativity of nitrogen heteroatoms. Additionally, nitrogen doping improved the material's morphology, porosity, and hydrophilicity, further enhancing its performance. To support this, we conducted a comparative photothermal performance assessment of rGO and N@rGO, exposing both materials to direct solar irradiation. Solar absorption and surface temperature increase, critical indicators of photothermal efficiency, were measured. As shown in Figure S1 (Supporting Information), N@rGO exhibited significantly higher solar absorption and surface temperature increase compared to rGO, which directly correlates to its superior desalination performance.

### 3.4 Photothermal Conversion Efficiency

The solar‐driven interfacial desalination performance of the evaporator is evaluated based on the mass of vapor generated and the solar‐to‐steam (photothermal) conversion efficiency. The photothermal conversion efficiency (η) is determined by calculating the ratio of the thermal energy stored in the generated vapor to the incoming solar flux, which can be computed using the relationship provided in Equation (1).^[^ ^54^ ^]^

(1) $$\eta =\frac{\dot{m}h_{LV}}{q_{i}}\times 100\%$$

where $\dot{m}$ refers to the net evaporation rate of water after subtracting the evaporation rate under dark conditions, h_LV_ refers to the total liquid‐vapor phase‐change enthalpy (sensible heat and latent heat), *q_i_* is incident solar power density, value of 1 kW m^−2^.

(2) $$h_{LV}=C\Delta T+h_{vap}$$

where C is the specific heat capacity of simulated seawater (3.993 J g^−1^ K^−1^), ∆T refers to the temperature rise of water, and *h_vap_* is the vaporization enthalpy at the surface of the porous macrostructure.

$\dot{m}$ is determined to be 1.94 kg m^─2^ h^─1^, h is the total enthalpy of sensible heat (294 kJ kg^─1^, from 24.1 to 54.5 °C with specific heat of 3.993 J g^─1^ K^─1^) and phase transition of seawater from liquid to gas at 54.5 °C (2371 kJ kg^─1^).

The photothermal conversion efficiency was determined to be ≈134%, which is a superb performance comparable to the values reported in other studies.^[^ ^54^, ^55^ ^]^ The observed high photothermal conversion efficiency is attributed to the interfacial desalination system's ability to recycle latent heat and retain the heat released from the surface, effectively minimizing heat loss and enhancing overall efficiency and water production.^[^ ^56^ ^]^ Additionally, the photothermal conversion efficiency exceeding 100% is accredited to the introduction of extra cold evaporation surfaces through the 3D structural design, which significantly harnesses environmental energy.^[^ ^57^ ^]^ Although these surfaces are not directly exposed to solar irradiation, they absorb heat from the surroundings, enhancing the evaporation process. Energy recycling from condensing water vapor further boosts the total energy input, synergistically resulting in extraordinary photothermal efficiency.^[^ ^58^, ^59^, ^60^, ^61^ ^]^

Specifically, minimizing the interface between the evaporator and water through structural design or incorporating cold surfaces to harness environmental energy significantly reduces heat loss. Designing specific water pathways for the evaporator to ensure a proper water supply is another effective measure to improve efficiency.^[^ ^57^, ^62^ ^]^ Bu et al.^[^ ^55^ ^]^ reported an evaporation rate of 3.27 kg m^2^ h⁻¹ and an evaporation efficiency of 194.4% under 1 sun illumination. This remarkable performance was achieved using a solar evaporator based on a 3D carbon fiber‐cotton (CFC) material. The innovative conical design of the 3D CFC‐Cone evaporator balances water transport and evaporation, optimizing energy distribution on the carbon fiber surface. The conical shape enhances light absorption through multiple reflections, while branched cotton bars and trunks serve as cooling surfaces to minimize heat loss. Similarly, Kim et al.^[^ ^56^ ^]^ demonstrated the efficient integration of 3D graphene networks (3DGNs) with wood pieces. The 3DGNs functioned as superior solar absorbers, while the wood provided excellent thermal insulation and facilitated water transport. Under 1 sun illumination, the composite achieved an impressive photothermal conversion efficiency of 91.8%, attributed to the insulating properties and water supply channels of the wood template. The high solar‐to‐thermal conversion efficiency of 3D N@rGO solar absorbers was attributed to their highly porous structure and broadband solar absorption across the visible to near‐infrared (UV–vis–NIR) region. The thermal insulation provided by the cellulose sponge in the system localized interfacial heating, preventing bulk heat dissipation into the water reservoir.^[^ ^63^ ^]^

Li et al.^[^ ^64^ ^]^ developed a COF/graphene hydrogel (CGH)‐based solar vapor generator featuring hydrophilic regions created by the covalent organic framework (COF)‐loaded reduced graphene oxide (COF@rGO) and hydrophobic regions formed by pure rGO. This design was achieved through the controlled deposition of sulfonic acid‐functionalized COF (COF‐SO₃H) onto rGO using hydrothermal synthesis. By systematically adjusting the ratio of hydrophilic and hydrophobic regions, the hydrogel exhibited improved light absorbance, optimized water content, a higher proportion of weakly bonded water, and reduced evaporation enthalpy. These features enabled exceptional solar‐vapor conversion performance, achieving a vapor generation rate of 3.69 kg m⁻^2^ h⁻¹ and a solar‐to‐vapor efficiency of 92% under 1 sun irradiation.

The management of high‐salinity streams, which arise from various sources such as oil and gas extraction, inland desalination concentrates, landfill leachate, and wastewater from industries like coal‐to‐chemical, textile, mining, and leather tanning, is increasingly recognized as a growing environmental concern. The direct discharge of these high‐salinity streams into surface waters poses a serious risk to ecosystems and the contamination of drinking and agricultural water supplies due to the extremely high salt concentrations and the widespread presence of pollutants.^[^ ^65^ ^]^ Desalination is becoming the preferred solution for handling hypersaline streams, offering notable environmental and economic benefits compared to existing methods.^[^ ^66^ ^]^ Thus, it is worth further investigating the solar‐driven desalination potential of the 3D photothermal material under hypersaline conditions.

**Figure 10:**
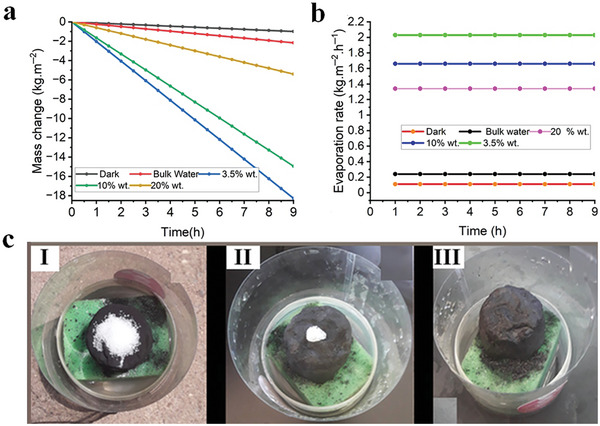
Mass change curves of water with increasing salinity a); and the corresponding evaporation flux under the natural sunlight in the real‐world solar riven desalination test of the various salinity b); salt resistance and self‐cleaning potential of the 3D N@rGO solar evaporator under extended duration c); at 0 h (I), 24 h (II), 48 h (III) carried out to test its long‐term, and stable water desalination performance under 1 sun solar irradiation (1 kW m^−2^).

In light of this, the solar‐driven interfacial desalination potential of the 3D solar absorber was evaluated at higher salinity levels (10 and 20 wt.%) under 1 sun solar irradiation, in darkness, and without the 3D solar absorbers as controls, to assess its ability to treat high‐salinity brine. As shown in **Figure** **10**a,b, the 3D N@rGO evaporator achieved evaporation rates of 2.05, 1.66, and 1.31 kg m⁻^2^ h⁻¹ for salinity levels of 3.5, 10, and 20 wt.%, respectively, under 1 sun solar irradiation. The evaporation rate for bulk water without the evaporator was 0.22, 0.2 kg m⁻^2^ h⁻¹ for the 10 and 20 wt.% salinity levels, respectively, slightly lower than the 0.24 kg m⁻^2^ h⁻¹ evaporation flux observed at the 3.5 wt.% salinity level. However, there was no significant change in the evaporation rates in the dark at 10 and 20 wt.% salinity levels, both being ≈0.11 kg m⁻^2^ h⁻¹.

The 3D photothermal material demonstrated efficient interfacial desalination performance in all salinity levels within the initial 7‐h period. However, after 7 h of desalination test, a gradual formation of salt crystals commenced at the 20% wt. salinity level. Over an extended desalination process of 21 h, the salt accumulation progressed, and the 3D evaporator with 20 wt.% salinity level was completely covered as portrayed in Figure S2 (Supporting Information). On the other hand, there was only slight salt accumulation observed on the 3D evaporator with 10 wt.% salinity level, the thinly salt crystals formed managed to be redissolved in the evening and returned to the bulk water through the mechanisms of convection and diffusion.^[^ ^67^ ^]^ This phenomenon demonstrated the self‐cleaning capability of the absorber, which can be attributed to the effective transfer of water through the microchannels, enabling a reversed flow of salt ions in accordance with the salt concentration gradient.^[^ ^67^ ^]^ However, the salt crystals that were cultivated on the 3D evaporator with a salinity of 20 wt.% were incapable of being redissolved and flowing back to the bulk water (as depicted in Figure S2a–c, Supporting Information). This occurrence might be linked to the elevated concentration of salt in the unevaporated bulk water that remains, which approaches the saturation level of salt in water at room temperature (35 wt.% NaCl).^[^ ^54^ ^]^ As a result, there is an extremely low concentration gradient between the bulk water and the salt ions present on the surface of the evaporator, thereby posing difficulties in terms of backwashing and dissolution. The progressive decline in the evaporation flux of the saline water with increasing salinity can be attributed to the reduction in the vapor pressure of water as salinity levels rise and reduced water activity.^[^ ^68^ ^]^ The high salt crystallization on the exterior surface of photothermal materials leads to a reduction in their ability to absorb light effectively. This, in turn, hinders the generation of steam and significantly impedes the process of solar steam generation, resulting in the lowest rate of evaporation.^[^ ^69^ ^]^ Hence the 3D solar evaporator demonstrated efficient self‐cleaning potential up to a salinity level of 10 wt.%. At this level, the tiny salt crystals that formed on its surface redissolved naturally during nighttime when the absence of a thermal gradient prevented capillary‐driven upflow of saline water to the evaporator's surface. However, as the salinity level increased to 20 wt.%, salt crystals began to form more prominently on the evaporator's surface. These crystals grew rapidly, eventually covering the evaporator and hindering its functionality. At this point, physical removal or prolonged flotation of the material in bulk water was necessary to enable the redissolution of the crystals and gradient‐driven backflow of salt ions. Hence in cases where significant salt accumulation was observed (e.g., at 20 wt.% salinity), the crystals were either scraped off manually after some time or allowed to redissolve naturally by leaving the evaporator floating in the absence of light, provided that adequate water was available. Sufficient water ensures capillary flow and facilitates the redissolution and reverse flow of salt ions.

### 3.5 Self‐Cleaning Performance of 3D N@rGO Solar Absorber

Salt crystallization is one of the most critical challenges limiting the stable performance of solar evaporators and salt resistance ability is an important index used to evaluate their long‐term stable performance. The 3D N@rGO solar absorber demonstrated high salt resistance and self‐cleaning capacity. The long‐term salt resistance and self‐cleaning potential of the 3D N@rGO solar absorber were further evaluated by placing 2 g of NaCl crystals on the surface of the solar absorber on a 3.5 wt.% salinity bulk water. The study demonstrated complete elimination of the salt accumulated over the 48‐h desalination study conducted with no trace of salt remaining on the solar evaporator, as presented in Figure 10c, which demonstrated the superb salt resistance and self‐cleaning capability of the 3D N@rGO. The porous morphology, adequate diffusion channels, and superhydrophilicity of the 3D evaporator ensured rapid water replenishment for continuous vapor generation as well as the diffusion and backflow of salt ions, therefore boosting the salt resistance potential of the solar evaporator.^[^ ^70^, ^71^ ^]^ The high photothermal conversion and interfacial desalination performance can be attributed to multidirectional mass and heat transfer as well as vapor removal, and high‐water transfer potential that ensures efficient water replenishment to the evaporative sites.^[^ ^70^ ^]^

### 3.6 Outdoor Solar Desalination Test

**Figure 11:**
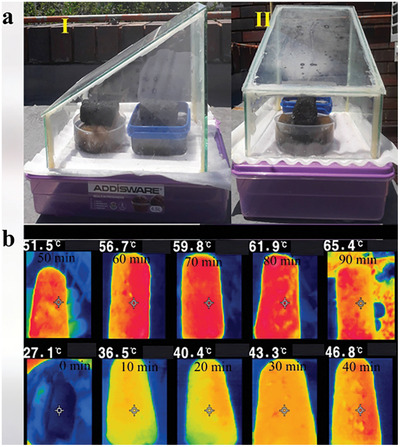
Modified solar still‐based solar‐driven interfacial desalination setup consisting of two 3D N@rGO solar absorbers laying on a cellulose insulation sponge floating on the saline water‐containing vessels. Small apertures have been drilled on the flat plate to let the condensed freshwater into the big plastic container underneath a); infrared images showing the temperature distribution during solar irradiation over a period of 90 mins b).

To assess the real‐world solar‐driven interfacial desalination potential of the 3D N@rGO evaporator, a prototype solar desalination setup with a modified single slope design was constructed. This setup consisted of double 3D N@rGO solar evaporators (each with a diameter of 4.0 cm and length of 7.0 cm), a vapor condenser, and a water collector underneath the saline water‐containing vessels (**Figure** **11**a I,II). The transparent glass covers the device, condenses the evaporated water, and subsequently converges freshwater generated to the water collector underneath through the apertures. The desalination device was placed on the rooftop of the environmental engineering and water utilization engineering building at the University of Pretoria (S25° 45′ 21″ E28° 13′ 51″), from February 23–25, 2024. The outdoor solar desalination experiment was carried out using simulated seawater of salinity level 3.5% wt. and took place from 9:00 am to 4:00 pm under natural sunlight with an average 8 h solar heat flux of ≈0.853 kw m^−2^ reaching up to 1.1 kW m^−2^ at around noon.^[^ ^72^ ^]^

Under natural sunlight irradiation, the dual 3D N@rGO evaporators generated water vapor, which is condensed on the transparent cover and collected in the plastic container (Figure 11a). On average ≈117 g of condensed water was collected daily, resulting in an average water evaporation rate of 2.61 kg m^−2^ h^−1^, based on an effective evaporation surface area of 56 cm^2^ over the 3‐day period monitored. In comparison to the desalination study conducted under simulated sunlight (1.0 kW m^−2^), which achieved an evaporation rate of 2.05 kg m^−2^ h^−1^, the outdoor test obtained an average evaporation rate of 2.61 kg m^−2^ h^−1^, indicating a 21.8% increase in the potential for freshwater generation.

**Figure 12:**
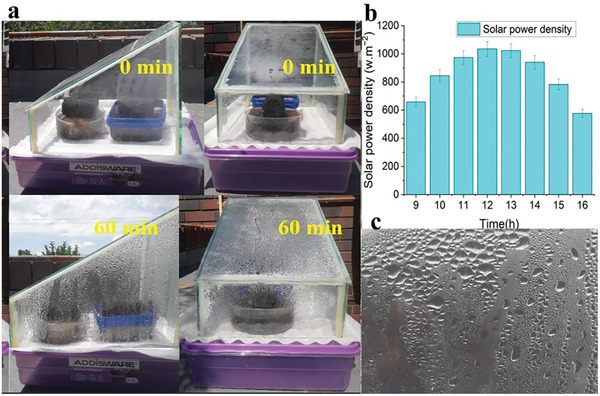
Typical vapor generation observation over a time of an hour from the side and front views of the solar‐driven interfacial desalination setup a); time‐dependent average solar power density over the three days collected from the online database from 9:00 am to 4:00 pm b); magnified portion showing condensate on the glass moving down into the freshwater collector c).

The 3D evaporator exhibited a rapid increase in surface temperature, reaching a maximum of 65 °C within an hour and a half, as portrayed in Figure 11b, demonstrating its remarkable photothermal efficiency. Unlike the solar‐simulated indoor experiment, the solar flux in real outdoor conditions fluctuates over time. At noon, the solar power density peaked at 1.1 kW m^−2^, as presented in **Figure** **12**b, resulting in a surface temperature response of up to 65 °C. This value is significantly higher than the temperature achieved in the indoor solar simulation test, where the solar simulator lens is positioned directly above the evaporation surface, with a maximum temperature of 54.5 °C under 1 sun. Conversely, the multidirectional solar absorption of the 3D structure allows for heat absorption from all directions, in contrast to the top‐directed solar simulation. This feature highlights excellent photothermal efficiency and surface temperature response. The exceptional photothermal performance observed in actual outdoor conditions can be attributed to all directional solar absorption coupled with multiple reflecting morphology of the 3D evaporator, the recovery of latent heat, and the collection of environmental energy from the surroundings, leading to a reduction in vaporization enthalpy.^[^ ^73^, ^74^ ^]^

The porous microstructure of the 3D surface reduces the diffuse reflection and enhances the light absorption potential, the invading light was reflected multiple times between the microporous channels, improving light absorption of the 3D evaporator.^[^ ^54^ ^]^ The superb photothermal performance of the 3D evaporator and the efficient heat management in the desalination set‐up demonstrated efficient freshwater generation potential, giving it a promising potential in tackling the urgent freshwater scarcity facing the globe. In addition, the scalability of the 3D N@rGO‐based solar‐driven desalination system is a significant factor to consider. The system can be scaled up by increasing the dimensions and array of solar evaporators accordingly. The salt rejection is determined by evaluating the reduction in salinity percentage between the collected freshwater and the feed using Equation (3).^[^ ^75^ ^]^

(3) $$Salt\;rejection\left(\%\right)=\left(1-\frac{\partial_{f}}{\partial_{i}}\right)\times 100\%$$

where ∂_*f*_ is the conductivity of the harvested water, and ∂_*i*_ is the conductivity of the bulk feed water. The concentration of ions in the water that was collected showed a significant reduction, reaching as low as 30 mg L^−1^ salinity (55 µS cm^−1^). This level is considerably lower than the salinity level recommended by the World Health Organization for drinking water, which is 200 mg L^−1^. The reduction in the harvested water's conductivity to 55 µS cm⁻¹, equivalent to a salt concentration of 30 mg L⁻¹, from the original feedwater with a salinity level of 3.5 wt.% and a conductivity of 52000 µS cm⁻¹, demonstrates a 99.7% salt rejection efficiency. The rooftop outdoor test provided an average daily freshwater yield of ≈21 kg m^−2^ at an 8‐h long desalination test carried out daily from 9 a.m. to 10 p.m. The freshwater generated, 21 L a day is equivalent to the minimum freshwater requirement capable of satisfying an average family size of two according to WHO.^[^ ^76^ ^]^ The outdoor freshwater yield reported in the current study is comparable to the freshwater yields achieved in similar 3D‐based solar‐driven interfacial desalination outdoor tests.^[^ ^54^, ^55^ ^]^

An ideal solar absorber should display broadband solar absorption across the entire solar spectrum, and the setup should minimize energy dissipation through radiation to the external environment or conduction to the bulk water. In the current study, the cellulose sponge served as an ideal insulator to protect heat dissipation and served as a water channel to transfer from the bulk to the porous 3D absorber. The high photothermal conversion efficiency of the 3D N@rGO observed in the current study can primarily be attributed to the elevated electrocatalytic activity and solar absorption performance, along with the high hydrophilicity and water transfer potential of the nitrogen‐doped 3D rGO solar absorber, as well as efficient thermal management placed. The introduction of nitrogen doping leads to a higher positive charge on a carbon atom adjacent to the nitrogen atoms, resulting in enhanced electrocatalytic activity and conductivity for efficient photothermal performance.^[^ ^52^ ^]^ These characteristics greatly facilitate the photothermal conversion of N‐doped graphene sheets, meeting the high demands of rapid solar evaporation.^[^ ^77^ ^]^ Notably, recent studies have shown that certain extrinsic features of chemically modified graphene exhibit significant potential in various applications, such as energy harvesting, supercapacitors, field‐effect transistors, and solar cells.^[^ ^78^ ^]^ The bonding of the N atom with a carbon framework introduces a defect in nearby sites due to the disparity in bond length and atomic size, consequently inducing a higher positive charge distribution on the adjacent carbon atoms. This phenomenon is attributed to the electron‐withdrawing ability of N, resulting in the enhancement of reactivity and electrocatalytic activity, ultimately boosting the electrocatalytic activity and electrical conductivity of the graphene and thereby increasing its photothermal performance.^[^ ^42^ ^]^

The hierarchically porous and hydrophilic structure of the 3D N@rGO evaporator offers it a significant advantage in replenishing water to the evaporation surface and facilitating two‐directional ion diffusion.^[^ ^36^ ^]^ Moreover, the ability to modify the electrical and optical properties of GO through chemical and thermal reduction processes has made GO‐based macrostructures a promising choice for achieving excellent light absorption and prominent photothermal performance.^[^ ^79^ ^]^ After reduction, the electrical conductivity of GO can be greatly improved and adjusted by manipulating the oxygen content in rGO. As a result, nitrogen‐doped reduced graphene oxide exhibits semiconductor behavior, with its electrical conductivity being adjustable through the control of oxygen levels and the presence of chemically active defective sites. This makes it a potential candidate for applications in photothermal conversion and solar desalination.^[^ ^79^, ^80^ ^]^ The 3D porous macrostructure assisted in maximizing solar absorption by enabling multiple reflections of incident light and minimizing energy loss, thereby contributing to the light absorption and evaporation efficiency of the evaporators.^[^ ^81^ ^]^ Besides, the larger water/air interface of the 3D macrostructure and lateral surface of the solar absorber is beneficial for more efficient vapor release and light absorption.

### 3.7 Costs of Materials and Energy Required for Scaling Up of the 3D N@rGO Solar Absorber

To synthesize 3D N@rGO with a top surface area of 0.0028 m^2^, 4.5 g of GO and 5 g of EDTA are required. Scaling up to produce 3D N@rGO with a total top surface area of 1 m^2^ necessitates proportional increases in these ingredients. As detailed in Table S1 (Supporting Information), the total material cost for producing 3D N@rGO with a total top surface area of 1 m^2^ is estimated to be $2237.

The energy costs for hydrothermal reduction and freeze‐drying have been estimated based on relevant process parameters and scale of production. According to Lucian and Fiori,^[^ ^82^ ^]^ a comparable process, hydrothermal carbonization, requires 160 kWh ton^−1^ of hydrochar, translating to ≈6.64 kWh for the hydrothermal reduction of GO to ≈2 kg of N@rGO. Additionally, medium‐sized freeze dryers consume 990–1500 W h^−1^, leading to an estimated energy usage of ≈30 kWh per load. Combining these processes, the total power consumption for producing 1 m^2^ of 3D N@rGO is estimated at 36.64 kWh. Given the South African electricity tariff of ZAR 3.292 per kWh ($ 0.184), the total energy cost amounts to $ 6.73. This indicates that energy costs are much lower compared to the expense spent on the chemicals, requiring more focus on minimizing material costs.

The initial production cost of 3D N@rGO is relatively high, largely due to the significant raw material demands of Tour's method for GO synthesis. However, the scalability of production can influence fabrication costs. Despite the upfront expense, N@rGO's exceptional durability and flexibility ensure a long service life, making it a valuable long‐term investment with low operating costs. Once installed, the system operates without additional running costs, making it especially advantageous for water‐scarce regions, such as deserts or areas with saline or brackish water but limited freshwater access. Its low operational and maintenance requirements, combined with negligible downtime, significantly enhance its cost‐effectiveness, outweighing the initial investment.

Efforts to reduce production costs including exploring alternative methods, such as employing wood‐based substrates coated with N@rGO are underway. This approach considerably lowers material and processing expenses while maintaining high performance. Although the initial production costs remain elevated due to the use of specific materials and chemicals, these are well justified by the system's extended lifespan, environmental benefits, and sustainability.^[^ ^83^ ^]^

Skuse et al.^[^ ^83^ ^]^ conducted an in‐depth study on the environmental benefits of GO‐based solar desalination systems, demonstrating their significant potential to reduce environmental impacts. Using life cycle assessment (LCA) modeling in GaBi v9.2 and applying the ReCiPe 2016 methodology, the study assessed impacts mitigated across 18 categories, including climate change potential, fossil fuel depletion potential, metal depletion potential, human toxicity (both cancer and non‐cancer effects), and ionizing radiation potential. Despite the high initial investment, the results highlighted the sustainability and long‐term viability of GO‐based desalination systems, emphasizing their capacity to mitigate environmental impacts and promote eco‐friendly water management solutions.

## 4 Conclusion

In this study, a highly porous 3D N@rGO solar absorber was developed that exhibited high solar absorption and superb photothermal conversion efficiency of ≈134% under 1 sun irradiation. Additionally, it demonstrated a high freshwater generation rate of 1.94 kg m^−2^ h^−1^ under 1 sun as well as a freshwater generation potential of 2.61 kg m^−2^ h^−1^ from simulated seawater over a 3‐day outdoor test. The high photothermal conversion potential can be attributed to the latent heat recycled from vapor condensation and the effective heat management system, which minimizes radiative and conductive heat losses. Nitrogen doping, employed as an effective strategy to create defects and regulate the electronic structure of graphene, significantly contributes to the high photothermal conversion efficiency and exceptional solar absorption performance. The highly porous and wrinkled morphology of the 3D N@rGO composite enables multiple reflections of incident light, minimizing energy loss and enhancing light absorption and evaporation efficiency in the evaporator. Furthermore, the solar absorber demonstrated efficient resistance to salinity levels up to 10 wt.%, owing to its high hydrophilicity and water transfer properties, which facilitate the backflow of salt ions into the bulk. This study demonstrates the promising potential of scalable 3D N@rGO in achieving remarkable photothermal conversion efficiency and freshwater generation from seawater as well as hypersaline water. The 3D solar absorber can be easily fabricated, making it suitable for large‐scale desalination and clean water generation, offering significant potential to address the freshwater shortage challenge faced by many countries.

## Conflict of Interest

The authors declare no conflict of interest.

## Supporting information



Supporting Information

## Data Availability

The data that support the findings of this study are available in the supplementary material of this article.
