# Supplementary material for: Synthesis and Evaluation of 3D Nitrogen Doped Reduced Graphene Oxide (3D N@rGO) Macrostructure for Boosted Solar Driven Interfacial Desalination of Saline Water
Source: Glob Chall. 2025 Feb 25;9(4):2400080. doi: 10.1002/gch2.202400080 (PMC12003209; doi:10.1002/gch2.202400080)
Supplement: Supplementary file 1 — Supporting Information [file GCH2-9-2400080-s001.docx]

**Synthesis and evaluation of** **nitrogen doped 3D-reduced graphene oxide nanosheets (3D N@rGO) potential application for solar driven interfacial desalination**

Fisseha A Bezza^a*^, Samuel A. Iwarere^a^ , Shepherd M. Tichapondwa^a^ . Hendrik G. Brink^a^ , Michael O.Daramola^a^, Evans MN Chirwa^a^

^a^ Water Utilization and Environmental Engineering Division, Department of Chemical Engineering, University of Pretoria, Pretoria 0002, South Africa

*fissehaandualem@gmail.com


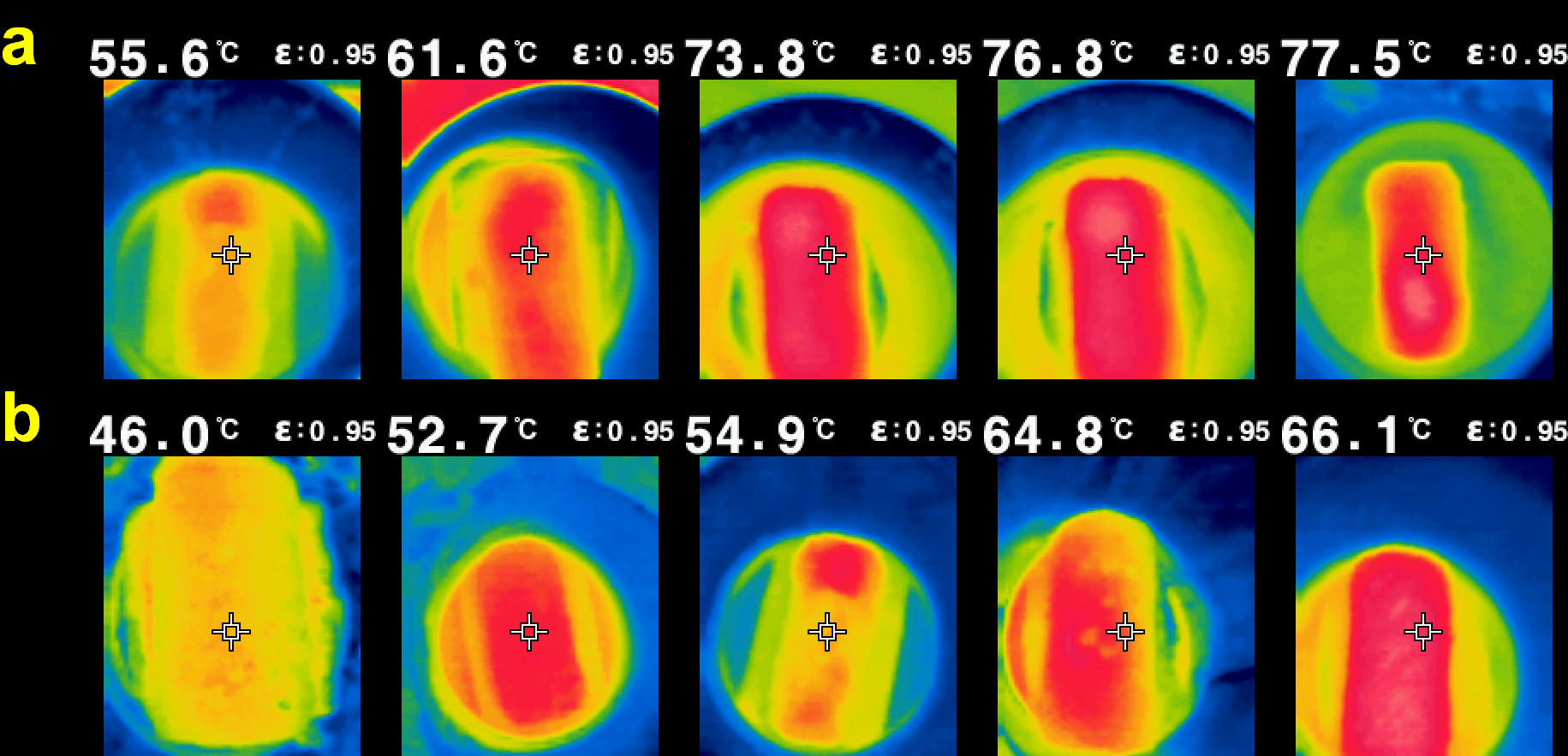


**Supplementary Figure 1.** Comparative evaluation of the solar absorption capabilities of reduced graphene oxide (rGO) and nitrogen-doped reduced graphene oxide (N@rGO), observed under direct solar radiation. The temperature response of both materials was monitored using an IR camera to assess their solar absorption efficiency.


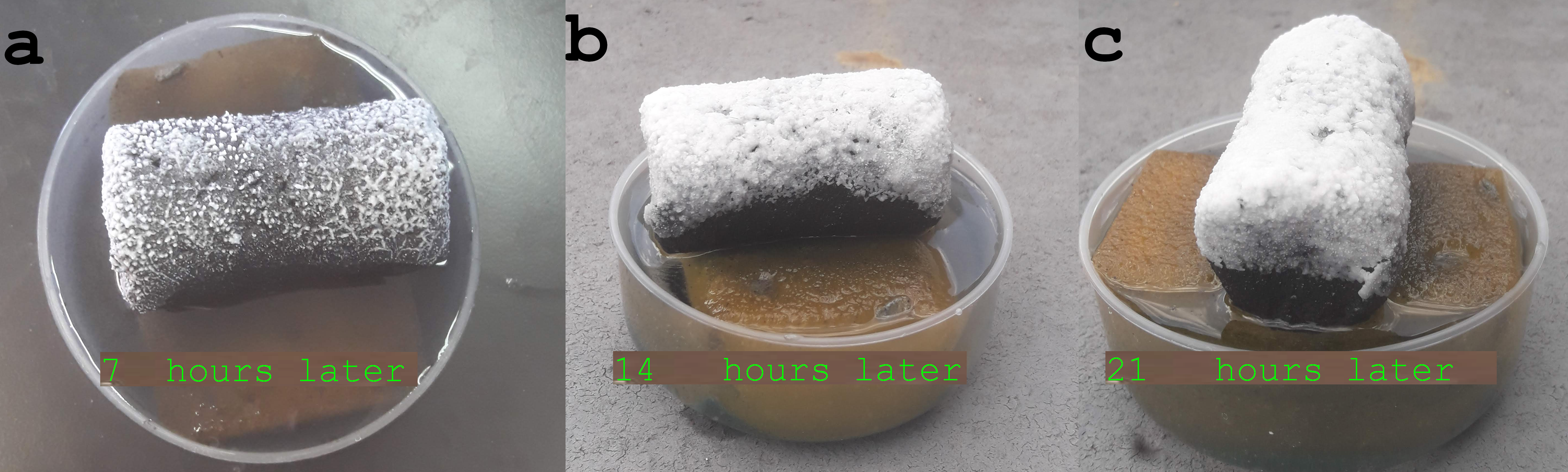


**Supplementary Figure 2**. Progressive salt accumulation on the 3D N@rGO solar absorber with 20% salinity level; after 7 hours (a), 14 hours (b), and 21 hours of solar driven interfacial desalination tests (c) under 1 sun solar irradiation.

The synthesis of 3D nitrogen-doped reduced graphene oxide (N@rGO) with a top surface area of 0.0028 m² requires 4.5 grams of graphene oxide (GO) and 5 grams of ethylenediaminetetraacetic acid (EDTA). Consequently, producing 1 m² of N@rGO requires 1605 grams of GO and 1786 grams of EDTA. According to Tour's method, 5 grams of GO are obtained from 3 grams of graphite nanoflakes. To scale up the production from 5 grams to 1605 grams of GO, a scaling factor of 321 is applied, meaning all ingredient quantities must be multiplied by 321. The detailed cost breakdown is as follows:

***Supplementary Table 1*** Summary of Chemicals and Costs for the production of 3D N@rGO with total top surface are of 1 m²

| **Chemical** | **Amount** | **Price (USD)** | **Supplier** |
| --- | --- | --- | --- |
| Graphite | 983.0 g | $65 | Sigma Aldrich |
| Potassium permanganate | 5778.0 g | $346 | Sigma Aldrich |
| Sulfuric acid | 109.2 L | $144 | Mining Chemicals SA |
| Phosphoric acid | 12.84 L | $960 | Sigma Aldrich |
| Hydrogen peroxide | 12.84 L | $267 | Sigma Aldrich |
| EDTA | 1786.0 g | $455 | Sigma Aldrich |
| **Total Cost** |  | **$2237** |  |
